# Breast implant causes allergic contact dermatitis or foreign body reaction?

**DOI:** 10.1080/23320885.2020.1810578

**Published:** 2020-09-03

**Authors:** Hilde M. Bosker, Jorrit B. Terra, Martin M. Stenekes

**Affiliations:** a Department of Dermatology, University Medical Center Groningen, University of Groningen, Groningen, The Netherlands; b Department of Plastic Surgery, University Medical Center Groningen, University of Groningen, Groningen, The Netherlands

**Keywords:** Allergic contact dermatitis, immediate breast reconstruction, Becker expander/breast implant, foreign body reaction

## Abstract

In this case report we describe a 55-year-old Caucasian female who had developed an itching, erythematous plaque on the right breast seven months after she received a permanent tissue expander. Topical corticosteroids had no effect upon which a capsulectomy was performed and the complaints disappeared.

The number of breast reconstructions with implants, after mastectomy, has increased over time. It is a safe method and results in significant benefits in body image, self-esteem, sexuality and quality of life [1–3]. A common cause for breast reconstruction failure (e.g. resulting in implant removal) is infection [4]. Other complications are seroma, capsular contracture, necrosis, hematoma, chronic pain and BIA-ALCL [5]. More rare examples of complications consist of hypersensitivity to various chemical compounds, contact allergies to rubber compounds or a benign inflammatory response elicited by silicone [6,7]. Here we describe a case where skin complications of a patient resolve after removing her permanent tissue expander, without any evidence for underlying causes.

## Case presentation

A 55-year-old Caucasian female had a medical history of ductal carcinoma *in situ* (DCIS) on her right breast. A mastectomy on the right side was performed in a nearby hospital, followed by an immediate reconstruction using a permanent tissue expander (Mentor Siltex^TM^ Contour Profile^TM^ Becker^TM^ 35 Expander/Breast Implant Cohesive II^TM^). No additional oncologic treatment was performed.

The expander was filled periodically by injecting saline solution and methylene blue through the distant fill port. After seven months the exact location of the fill port could not be determined anymore, and several attempts were made before succeeding. The next day the patient developed an itching, erythematous plaque on the lateral side of the right breast, caudally to the injection site. There were no systemic symptoms. The patient had no prior history of atopic dermatitis or contact allergies. The diagnosis irritant contact dermatitis was determined and treatment with medium potency topical steroids was initiated.

**Figure 1.**
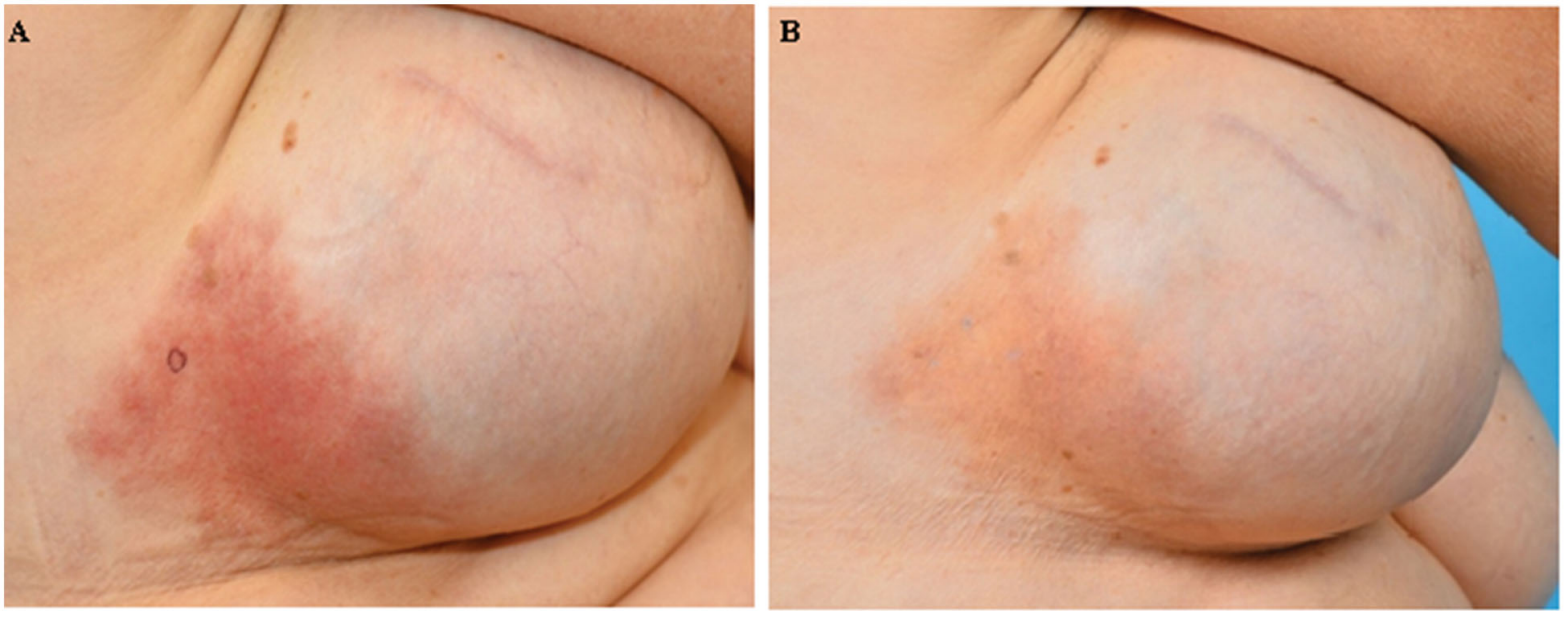
(A) The first clinical presentation where a moderate bordered, nummular erythematous to brown macule can be seen with pityriasiform squamae. (B) The clinical presentation after 3 months of topical therapy.

One year after the reconstruction the patient was referred to our outpatient clinic with persisting complaints of erythema and itch. The clinical findings consisted of a moderate bordered, nummular erythematous to brown macule with fine bran-like (pityriasiform) squamae (see Figure 1(a)). No urticaria or blisters were seen. There were no signs of induration, sclerosis or infection.

**Figure 2.**
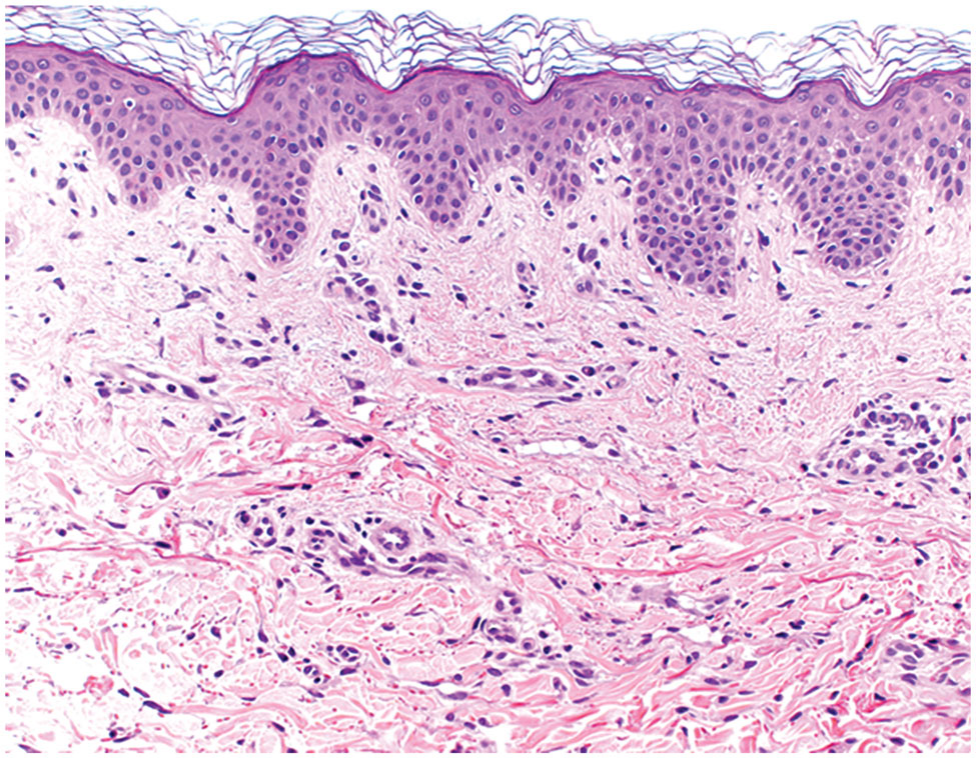
The histopathology with a superficial and deep perivascular dermatitis consisting of mainly lymphocytes and plasma cells.

Our differential diagnosis consisted of erythema chronicum migrans, morphea, allergic contact dermatitis and a granulomatous (foreign body) reaction. An ultrasound showed nothing unusual. Laboratory results showed e.g. erythrocyte sedimentation rate (ESR; 40), Hb (8.4), leukocytes (6.4) and alanine aminotransferase (ALAT; 19). The biopsy showed a superficial and deep perivascular dermatitis consisting of mainly lymphocytes and plasma cells (see Figure 2). The polymerase chain reaction (PCR) on the fresh biopsy was negative for Borrelia as was Borrelia serology. Furthermore, the safety sheets (SDS) of the permanent tissue expander were requested and patch tests were performed with our extended European Baseline series, cosmetics series, fragrance series, metal series, plastic glues series and the acrylates series. The patient only reacted positive to hydroperoxides of limonene which was considered to be irrelevant.

**Figure 3.**
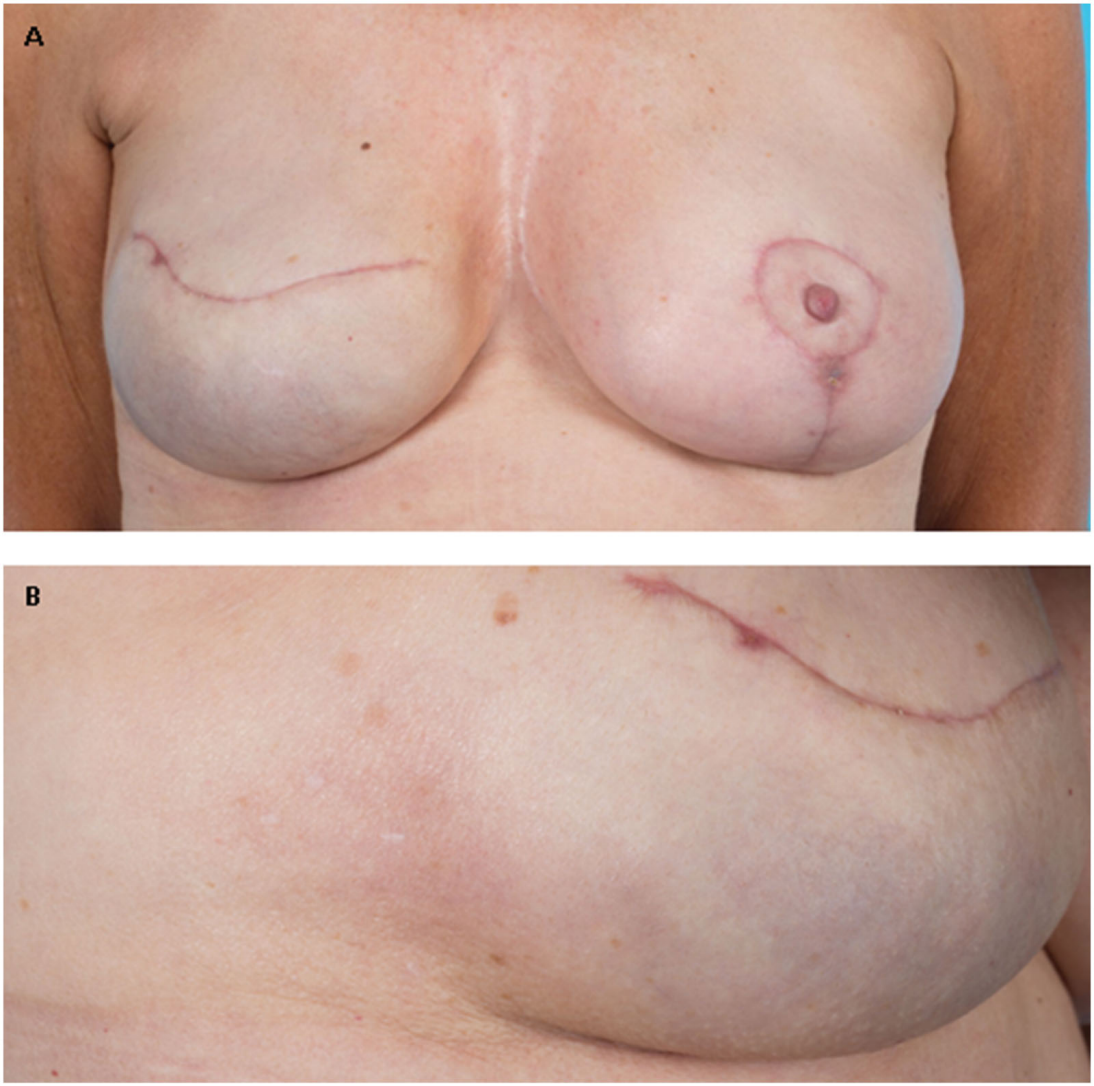
(A) The clinical presentation after removing the expander. (B) A detail photo where a subtle brown macula can be seen.

Since the skin complaints started a day after the difficulties finding the fill port, it seems to be related. Therefore, our work diagnosis was a foreign body reaction to (components of) the permanent tissue expander. Topical therapy was started, which reduced the itch/erythema (clobetasol cream, triamcinolone and tacrolimus 0.1% ointment, see Figure 1(b)). The medical staff of the expander manufacturer advised to start levoceterizin 5 mg daily, which seemed to reduce the itch slightly. However, while the skin symptoms were reduced, the patient still suffered from discomfort because of a Baker grade 3 capsular contracture and she was not content with the esthetic result of the implant. Eventually, 2 years after onset of the symptoms, a capsulectomy was performed together with a change of the expander with a silicone implant (Natrelle^TM^ Style 410MF) together with a symmetrizing mastopexy on the contralateral side. During surgery the expander was found to be intact. Within a few weeks all the symptoms disappeared, even after quitting the corticosteroid ointments (see Figure 3).

## Discussion

In this case report we present a patient with a permanent tissue expander in her right breast who developed an itching erythematous plaque after seven months. The skin complaints started the day after a visit to the outpatient clinic where the fill point could not be found easily. Eventually the problems resolved after removing the permanent tissue expander.

In this paper we stated that the most common cause for breast reconstruction failure is infection. In this specific case there were no clinical (or systemic) signs of infection. However the ESR was slightly increased this can be seen in any inflammatory process, for example a foreign body reaction [8]. Whenever there would be an acute infection one would expect increased leukocytes, lowered Hb and increased ALAT. Unfortunately there was no CRP determined, which is a limitation of this paper. A last possible option would be a subclinical infection but the histopathology of the biopsy showed no neutrophil granulocytes at all which is expected in a bacterial infection. Therefore we excluded an infection as underlying cause.

Previous research showed that silicone is a chemically stable compound but also, that they are capable of inducing an antigen-specific lymphocyte-medicated response to the silicone gel like a type IV hypersensitivity reaction [7,9]. When a patient has had a prior history of reactions to adhesives the possibility of a type IV hypersensitivity reaction to a silicone implant increases. Histologically lymphoid cells and granuloma are present on the implant capsule. In our patient the histology of the removed capsule with expander showed granulation tissue, fibrosis and an infiltrate consisting of lymphocytes, plasma cells, eosinophil en neutrophil granulocytes. This reaction can be seen as a reactive inflammatory process to the foreign-body. Since the histological findings were not specific and our patient did not had a prior history of reactions to adhesives, the type IV reaction seems to be unlikely.

A hypersensitivity reaction is possible when there is contamination with sensitizers such as rubber of metal compounds during manufacture [10]. Since we excluded contact allergy to rubber and metal this does not seems to be relevant here.

Since the symptoms developed one day after the broaching of the metal valve failed seven times consecutively the suspicion of a relation between this procedure was high. However, the broach site was on the upper outer quadrant of the right breast whereas the exposed site was more to the caudal side. A possible explanation for this discrepancy is gravity. Furthermore the question is which fluid would have caused the skin reaction since the fill fluid consisted of methylene blue and saline. When the methylene blue was concerned in this matter the skin would have turned blue as well, which was not the case. Also the fact that saline would give such a reaction is unlikely, unless it was contamination with micro parts (e.g. plastic).

In summary we have presented a woman with a permanent tissue expander who developed an itching erythematous plaque after seven months. Although no underlying cause was found the implant was removed because the patient continued suffering from her symptoms. After removing the implant her complaints disappeared within a week. However we do not have a solid explanation for this causal connection, our work hypothesis is a foreign-body reaction without granulomas. Furthermore, this case shows that replacing the implant can solve the symptoms. It can be worth performing in specific cases where no other underlying cause can be ascertained. .
